# Identifying stigmatizing and positive/preferred language in obstetric clinical notes using natural language processing

**DOI:** 10.1093/jamia/ocae290

**Published:** 2024-11-21

**Authors:** Jihye Kim Scroggins, Ismael I Hulchafo, Sarah Harkins, Danielle Scharp, Hans Moen, Anahita Davoudi, Kenrick Cato, Michele Tadiello, Maxim Topaz, Veronica Barcelona

**Affiliations:** School of Nursing, Columbia University, New York, NY 10032, United States; School of Nursing, Columbia University, New York, NY 10032, United States; School of Nursing, Columbia University, New York, NY 10032, United States; Icahn School of Medicine, Mount Sinai, NY 10029, United States; Department of Computer Science, Aalto University, Espoo 02150, Finland; VNS Health, New York, NY 10017, United States; School of Nursing, University of Pennsylvania, Philadelphia, PA 19104, United States; Center for Community-Engaged Health Informatics and Data Science, Columbia University Irving Medical Center, New York, NY 10032, United States; School of Nursing, Columbia University, New York, NY 10032, United States; School of Nursing, Columbia University, New York, NY 10032, United States

**Keywords:** natural language processing, electronic health records, health communication, bias, nursing informatics

## Abstract

**Objective:**

To identify stigmatizing language in obstetric clinical notes using natural language processing (NLP).

**Materials and Methods:**

We analyzed electronic health records from birth admissions in the Northeast United States in 2017. We annotated 1771 clinical notes to generate the initial gold standard dataset. Annotators labeled for exemplars of 5 stigmatizing and 1 positive/preferred language categories. We used a semantic similarity-based search approach to expand the initial dataset by adding additional exemplars, composing an enhanced dataset. We employed traditional classifiers (Support Vector Machine, Decision Trees, and Random Forest) and a transformer-based model, ClinicalBERT (Bidirectional Encoder Representations from Transformers) and BERT base. Models were trained and validated on initial and enhanced datasets and were tested on enhanced testing dataset.

**Results:**

In the initial dataset, we annotated 963 exemplars as stigmatizing or positive/preferred. The most frequently identified category was marginalized language/identities (*n* = 397, 41%), and the least frequent was questioning patient credibility (*n* = 51, 5%). After employing a semantic similarity-based search approach, 502 additional exemplars were added, increasing the number of low-frequency categories. All NLP models also showed improved performance, with Decision Trees demonstrating the greatest improvement (21%). ClinicalBERT outperformed other models, with the highest average F1-score of 0.78.

**Discussion:**

Clinical BERT seems to most effectively capture the nuanced and context-dependent stigmatizing language found in obstetric clinical notes, demonstrating its potential clinical applications for real-time monitoring and alerts to prevent usages of stigmatizing language use and reduce healthcare bias. Future research should explore stigmatizing language in diverse geographic locations and clinical settings to further contribute to high-quality and equitable perinatal care.

**Conclusion:**

ClinicalBERT effectively captures the nuanced stigmatizing language in obstetric clinical notes. Our semantic similarity-based search approach to rapidly extract additional exemplars enhanced the performances while reducing the need for labor-intensive annotation.

## Introduction

Stigmatizing language refers to language that unintentionally or intentionally conveys potentially harmful or discriminatory meanings, perpetuating socially constructed power dynamics.^1^ Stigmatizing language can convey implicit and/or explicit bias towards patients, particularly for marginalized populations.^1^^,^^2^ Bias in healthcare contributes to health disparities, including unequal care in terms of the number and quality of clinician-patient interactions.^3^^,^^4^ Bias also hinders the development of positive patient-clinician relationships and contributes to medical mistrust, which have been associated with disengagement and dissatisfaction in healthcare, exacerbating health disparities.^2^^,^^5^ Due to the known disparities in pregnancy and birth-related health outcomes among perinatal populations,^6^ it is critically important to identify stigmatizing language to prevent bias and disparities in obstetric care settings. Despite that, the existing body of research around stigmatizing language is limited and primarily focuses on general medicine populations, which may miss discipline-specific stigmatizing language.^7^

Clinical notes in electronic health records (EHRs) represent a rich data that often contain stigmatizing language.^7^ However, about 80% of EHR data is stored in an unstructured format, making it difficult to analyze using traditional analytic approaches.^8^ Recent advances in natural language processing (NLP) have enabled the analysis of this large text data to identify critical health concepts,^9^ such as stigmatizing language. NLP can efficiently process large unstructured text data from EHR, automatically extracting and categorizing text data into more meaningful information for clinicians.^10^ NLP is a powerful method designed to understand context, sentiment, and semantic of human language within the text,^11^ making it particularly suitable for identifying stigmatizing language which is often highly nuanced and contextual.^2^ While emerging studies have used NLP to identify stigmatizing language, they often rely on rule-based NLP models with keyword searches based on predetermined list of terms, limiting to capture the full spectrum of nuanced stigmatizing language.^12^^,^^13^

Our research team is pioneering the work on identifying stigmatizing language in obstetric care settings using clinical notes in EHR from hospital birth admissions. We piloted a NLP study to identify 2 broad categories of stigmatizing language: marginalized and power/privilege language.^13^ In this pilot study, marginalized language broadly referred to mentions of less socially desirable characteristics, negative connotations, or clinician’s disapproval. Examples included “patient denies illicit drug use” and “patient states baby will sleep in their bed. [social work] intern discussed though this may be cultural, it is important for baby to have his own bed.”^12^ Power/privilege language broadly referred to more socially desirable characteristics that may reflect signs of clinician’s approval,^14^ such as “patient reports having a nurturing marriage” and “husband is a neurosurgeon.”^13^ These previous studies provided a conceptual basis for stigmatizing language categories and a methodological foundation for further NLP model development. However, our pilot NLP study focused on only 2 broad categories of stigmatizing language. We conducted additional qualitative content analyses and further refined these 2 broad categories into multiple subcategories that describe more nuanced and specific stigmatizing language used in the current study. To capture the full spectrum of stigmatizing language more accurately, developing more advanced NLP models to identify and distinguish specific subcategories is essential.

In the current study, we first conducted human annotation of EHR notes to develop a labeled gold standard dataset. We sought to identify additional, refined stigmatizing language categories including preferred/positive language. We trained and tested various NLP models to determine the best performing model. We employed traditional classification models, such as Support Vector Machine (SVM) and Decision Trees, and advanced NLP models, ClinicalBERT (Bidirectional Encoder Representations from Transformers) and BERT base, that may be advantageous in identifying more nuanced language categories. In addition, we found limited exemplars of the new stigmatizing language categories from initial human annotation, which would challenge comprehensive NLP system training. Having scant training data is a common issue in NLP model development for specialized domains and tasks where initial human labeled training data are limited.^15^ To overcome this issue, we employed a streamlined approach based on semantic similarity to search and extract additional exemplars in clinical notes to enhance the performance of NLP models.

## Methods

### Data and study population

We used EHR data from patients >20 weeks’ gestation who were admitted for labor and birth at 2 urban hospitals in the Northeast United States in 2017. The full dataset included 742 503 notes, encompassing 556 different note types. All clinical notes recorded in the EHR during the inpatient stay were considered eligible. Clinical note types that did not contain substantive clinician narrative texts describing patient assessments or impressions were excluded, such as medication orders, transfer notes, and template-based statements about procedures or operations. Seven clinical note types were included in the current study (obstetric postpartum note, obstetric admission note, obstetric triage note, anesthesia resident note, miscellaneous nursing note, social work initial assessment, and initial nutrition assessment). These narrative clinical notes were preprocessed and prepared for analysis. The current study followed the ethical standards and received the Institutional Review Board approval from Columbia University Medical Center (AAAT9870).

### Human annotation of clinical notes

We randomly sampled at least 100 notes from each of the 7 note types to generate a human-annotated gold standard dataset. Four annotators with expertise in qualitative research and/or clinical nursing independently annotated clinical notes following an established codebook and procedure. The codebook was created based on iterative inductive-deductive content analysis and is published in detail elsewhere.^12^ Two annotators reviewed and annotated the same clinical notes to ensure the reliability of the annotation. Any disagreement was resolved through iterative discussions among annotators and with the entire research team. Annotators manually identified and labeled *exemplar* sentences that contained stigmatizing and positive/preferred language within the clinical notes. These exemplars typically span between 1 and 3 sentences and were used to develop the NLP models. Annotators ensured these exemplars were from the free-text section of clinical notes by carefully examining the writing style, format, and location of the texts. For example, “obese” or “obesity” in the structured sections of the note displayed a uniform language and format across different notes of the same note type. Conversely, documentations in the free-text sections are more individualized and distinct, conveying specific details and nuanced information about the patient rather than generic diagnosis (eg, obese with specific body mass index number that is applicable to the specific patient).

### Extracting additional exemplars to enhance training dataset

We aimed to improve the overall performance of the NLP models by increasing the number of exemplars, particularly for the categories with scant exemplars, in a less manually intensive way. We developed and employed a new approach based on semantic similarity search to expand the initial dataset of human-annotated exemplars while limiting the required manual labor of reading through and annotating the clinical notes from scratch. This new approach uses initial human-annotated exemplars as queries. Then, it searches and recommends new exemplars that are likely candidates from a larger dataset of unused clinical notes. In turn, the human annotators can relatively quickly review the recommended candidates and verify whether or not they are relevant exemplars of stigmatizing language.

To search for additional exemplars that were similar to the initial human-annotated exemplars, a sentence-transformer model was used (“multi-qa-distilbert-cos-v1”)^16^ to first encode exemplars into fixed size vectorized embeddings.^17^ Semantic similarity between exemplars (queries and candidates) were calculated using the cosine similarity metric applied to their associated embedding representations. The FAISS library was used to efficiently perform similarity searches.^18^ We included clinical notes that were unused for human annotation but were from the same note types. We preprocessed these notes by extracting the free-text portions using regular expressions and performed tasks such as removing special characters (eg, &quot;), handling abbreviations (eg, “pt.”), and normalizing text case. We converted the sentences into a similar format as the exemplars in the initial human-annotated (gold standard) dataset by forming them into single, double, or triple consecutive sentences to be prepared for the sentence transformer.

We randomly selected 250 human-annotated exemplars, representing a diverse range of language categories, to be used as queries. For each query (for each of the 250 exemplars), we retrieved the top 5 similar exemplars with the highest cosine similarities (resulting in 1250 exemplar candidates). Four annotators checked if these exemplar candidates were relevant. This approach yielded 502 additional exemplars that contain stigmatizing and positive/preferred language. The set of initial human-annotated exemplars were referred to as the initial dataset (IN) and the additional exemplars as the expanded dataset (EX). The combined set of initial and additional exemplars were referred to as the enhanced dataset (EN).

### Developing NLP models

**Figure 1.**
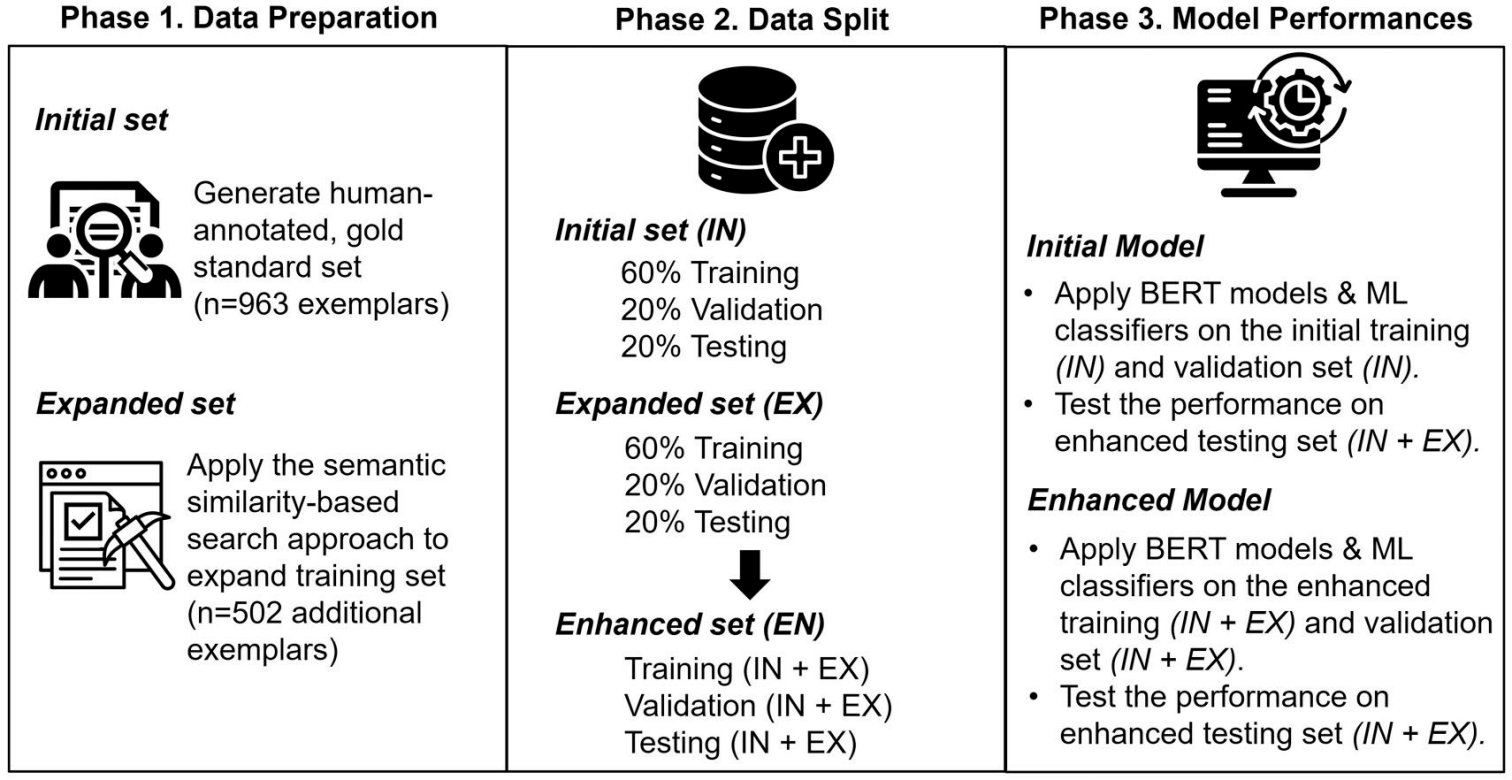
Overview of approach. Abbreviations: BERT, Bidirectional Encoder Representations from Transformers; ML, Machine Learning.

An overview of this study’s approach is presented in Figure 1. In preparing and designing our model, the initial dataset was compiled, including a balanced, equal number of positive and negative case exemplars (*N_in_* = 1926). We also added an equal number of negative case exemplars (text where stigmatizing language does not appear) for the expanded dataset (*N_ex_* = 1004). The negative case exemplars were randomly sampled from clinical notes not flagged for stigmatizing content, ensuring their length and context were analogous to the positive case exemplars.

Both initial and expanded datasets were stratified into training (60%), validation (20%), and testing (20%) datasets, maintaining category distribution to preserve data integrity. The split datasets were combined to form final enhanced training (*n_in+ex_* = 1757), validation (*n_in+ex_* = 586), and testing datasets (*n_in+ex_* = 587). As a general approach, we used the training and validation datasets to train the models and tested the performance on the enhanced testing dataset. We performed these tasks for both models trained on the initial and enhanced datasets to compare the performances before and after augmenting the training with additional exemplars.

We employed and trained the following NLP models: SVM, Decision Trees, Random Forests, ClinicalBERT,^19^ and BERT base.^20^ For the first 3 NLP models (traditional classifiers), we used the term frequency-inverse document frequency (TF-IDF) for feature generation and chi-squared feature selection.^21^^,^^22^ We combined the training and validation datasets into a single dataset, which we used to perform stratified 5-fold cross-validation with sample weights was implemented to ensure equitable evaluation and address class imbalances. Within each fold, Bayesian hyperparameter optimization was conducted to determine the optimal settings for the classifiers. Following tuning, the models were retrained on the entire training dataset. The trained model was then evaluated against the enhanced testing dataset ($n_{e}$ = 587) using bootstrap resampling with 1000 iterations, generating multiple resampled datasets to estimate the variability and stability of the model’s performance metrics.^23^^,^^24^ We calculated the bias-corrected and accelerated (BCa) confidence intervals for performance metrics across these bootstrap samples. The BCa method adjusts for both bias and skewness in the bootstrap distribution, providing more accurate confidence intervals.^25^ Additionally, we used the Wilcoxon signed-rank test to assess statistically significant differences between the performance metrics of initial and enhanced models (see Table S1).^26^

ClinicalBERT and BERT base were modified for our task. BERT base is a pre-trained language model on a large collection of texts from Wikipedia and books.^20^ ClinicalBERT is a domain-adapted model that is initialized on BERT base and further trained on unstructured clinical texts from a tertiary hospital setting similar to our data.^19^ We added a linear classification layer to each model, enabling the categorization of stigmatizing language. Gradient clipping was applied to prevent exploding gradients, setting the gradient norm cap at 1.0.^27^ Hyperparameter tuning was performed using the Optuna library^28^ to refine the models’ performance to maximize evaluation accuracy. The final models were trained with the best hyperparameters identified (learning rate = 3e-5, batch size = 16, epoch = 4), ensuring effective utilization of the models’ contextual knowledge to enhance predictive performance in clinical applications.

## Results

### Human annotation of initial gold standard dataset

We annotated 1771 clinical notes before reaching data saturation, where no new emerging language categories were identified. All language categories and their descriptions are shown in Table 1. Of the 1771 notes, 754 notes contained stigmatizing and positive/preferred language. Some of the notes contained language belonging to 2 or more categories. In total, 963 exemplars were annotated as stigmatizing or positive/preferred (Table 2). Most of these exemplars were stigmatizing (*n* = 757, 78.60%), while about 21% were positive/preferred language (*n* = 206). The 3 most frequently identified stigmatizing categories were marginalized language/identities (*n* = 397, 41.23%), difficult patient (*n* = 147, 15.26%), and power/privilege language (*n* = 92, 9.55%). Less than 100 exemplars were identified for unilateral/authoritarian decisions (*n* = 70, 7.27%) and questioning patient credibility (*n* = 51, 5.3%).

**Table 1.** Language categories and their descriptions.

Categories	Descriptions	Exemplars
Marginalized language/identities	Documentations of social and behavior factors that could contribute to marginalization for a specific group of people with what clinician perceived to be undesirable.	“she does not use drugs or smoke but drinks 6× week” “Patient is a 32yo [year old] Dominican unmarried unemployed female.” Restating for emphasis that is already in the checklist/form data or unnecessary patient descriptor: “toxic habits,” “financially supports self,” “teen mother,” “late registrant,” and “obesity.”
Difficult patient	Nonadherence, noncompliance, or refusal of care and services. Patient behaviors that are not in line with provider expectations.	“poor effort with pushing” “patient complaining of lightheadedness” “FOB [father of baby] is 23 yo [year old] unemployed and not as involved as he should be.”
Power/privilege	Power and privilege identities describing psychological or social-ecological status.	“reports nurturing marriage” “private pt of mine at 39 + 6 wks [weeks] with multiple episodes of emesis this AM”
Unilateral/authoritarian decisions	Supports clinician’s authority over patients, upholding hierarchy, centering clinicians, not patients.	“intolerant of vaginal exam” “SW [social worker] has advised pt [patient] that if there continues to be yelling in room, ACS [adult and child services] will need to be contacted.”
Questioning patient credibility	Disbelief in the patient’s report of health history or status.	“unsure if patient telling the truth” “pt [patient] denied any other DV [domestic violence] incidents and was adamant that relationship with spouse was healthy.”
Positive/preferred language	Describes birthing person exercising autonomy around birth. Uses words that convey patient’s point of view objectively.	“patient desires epidural prior to starting induction with Pitocin.” “on exam patient endorses numbness across entirety of bilateral buttocks” “amniocentesis was offered and recommended and the patient declined stating she would not terminate for a child with Down's syndrome.” “Pt [patient] declines epidural at this time- trying to deliver without analgesia.”

**Table 2.** Proportion of exemplars across language categories.

	Initial exemplars		Expanded exemplars		Final enhanced exemplars	
Categories	n	%	n	%	n	%
**Stigmatizing language**	757	78.61	298	59.36	1055	72.01
Marginalized language/identities	397	41.23	129	25.70	526	35.90
Difficult patient	147	15.26	91	18.13	238	16.25
Power/privilege	92	9.55	22	7.37	129	8.81
Unilateral/authoritarian decisions	70	7.27	37	4.38	92	6.28
Questioning patient credibility	51	5.30	19	3.78	70	4.78
**Positive/preferred language**	206	21.39	204	40.64	410	27.99
**Total**	963	100	502	100	1465	100

### Expanded exemplars to enhance training dataset

Next, we added 502 exemplars to enhance the training dataset (Table 2). About 59% of the expanded exemplars were categorized as stigmatizing language (*n* = 298). After adding the expanded exemplars, the final enhanced dataset had higher numbers for all language categories. In the initially low count categories, power/privilege and unilateral/authoritarian decisions had less than 100 exemplars in the initial dataset, which increased to ≥100 or close to 100 in the final enhanced dataset. The number of exemplars in the questioning patient credibility category also increased by about 37% in the final enhanced dataset.

### NLP model performances

The average performance scores of all 5 models are presented in Table 3. The best-performing initial models were BERT models, with an average F1-score of 0.75 for BERT base and 0.73 for ClinicalBERT. SVM was the best-performing initial model among the traditional classifiers, resulting in an average F1-score of 0.68. Decision Trees performed sub-optimally with average F1-scores of 0.56.

**Table 3.** Average performance of all models.

	Initial model			Enhanced model		
	F1	Precision	Recall	F1	Precision	Recall
ClinicalBERT	0.73	0.77	0.71	0.78	0.77	0.80
BERT base	0.75	0.77	0.75	0.77	0.76	0.78
SVM	0.68	0.72	0.66	0.76	0.79	0.75
Random Forest	0.67	0.73	0.62	0.76	0.81	0.72
Decision Trees	0.56	0.57	0.58	0.68	0.73	0.66
**Macro average**	**0.68**	**0.71**	**0.66**	**0.75**	**0.77**	**0.74**

*Note.* The macro average of all language categories for each model was reported.

Abbreviations: BERT, Bidirectional Encoder Representations from Transformers; SVM, Support Vector Machine.

After augmenting the initial dataset with expanded exemplars, all NLP models showed improved performance in the final enhanced model. The average F1-scores increased from 0.68 to 0.75 in the enhanced model, representing an average improvement of about 10%. ClinicalBERT was the final best-performing enhanced model with an average F1-score of 0.78, an increase of 0.05 from the initial score (7% improvement). Decision Trees, which had the lowest initial performance, showed the greatest improvement; the average F1-score increased from 0.56 to 0.68 (about 21% improvement). Initial models that performed sub-optimally with average F1-scores below 0.7 (SVM, Random Forest, and Decision Trees) achieved average F1-scores close to 0.7 or higher in the enhanced model.

The detailed performances of all 5 models are presented in Table 4. Most language categories consistently improved using the enhanced dataset across all 5 NLP models. For example, F1-scores increased from the initial to the enhanced model for questioning patient credibility and positive/preferred language categories across all 5 NLP models. Difficult patient and unilateral/authoritarian showed improvement in all 4 NLP models except for BERT base. Marginalized language/identities categories showed improvements for SVM, Random Forest, and Decision Trees.

**Table 4.** Detailed NLP model performances.

	Initial model														
	ClinicalBERT			BERT base			SVM			Random Forest			Decision Trees		
	F1 (95% CI)	Precision (95% CI)	Recall (95% CI)	F1 (95% CI)	Precision (95% CI)	Recall (95% CI)	F1 (95% CI)	Precision (95% CI)	Recall (95% CI)	F1 (95% CI)	Precision (95% CI)	Recall (95% CI)	F1 (95% CI)	Precision (95% CI)	Recall (95% CI)
Marginalized language/identities	0.90 (0.85-0.93)	0.94 (0.89-0.98)	0.86 (0.78-0.92)	0.92 (0.88-0.96)	0.93 (0.87-0.97)	0.92 (0.86-0.96)	0.91 (0.86-0.94)	0.99 (0.97-1.00)	0.84 (0.76-0.90)	0.82 (0.75-0.87)	0.99 (0.95-1.00)	0.70 (0.60-0.77)	0.70 (0.62-0.77)	0.91 (0.84-0.97)	0.57 (0.47-0.65)
Difficult patient	0.76 (0.65-0.86)	0.94 (0.84-1.00)	0.65 (0.48-0.77)	0.75 (0.63-0.83)	0.83 (0.70-0.92)	0.69 (0.53-0.79)	0.47 (0.36-0.58)	0.44 (0.34-0.54)	0.52 (0.38-0.65)	0.43 (0.31-0.53)	0.42 (0.31-0.53)	0.44 (0.31-0.58)	0.30 (0.20-0.40)	0.28 (0.19-0.37)	0.33 (0.21-0.46)
Power/privilege	0.77 (0.61-0.87)	0.73 (0.55-0.86)	0.82 (0.55-0.91)	0.72 (0.58-0.84)	0.65 (0.50-0.79)	0.82 (0.59-0.91)	0.80 (0.67-0.91)	0.89 (0.76-1.00)	0.73 (0.59-0.91)	0.70 (0.54-0.82)	0.79 (0.64-0.94)	0.64 (0.46-0.77)	0.56 (0.41-0.71)	0.55 (0.39-0.70)	0.59 (0.41-0.82)
Unilateral/authoritarian	0.66 (0.46-0.80)	0.77 (0.56-0.93)	0.59 (0.32-0.73)	0.68 (0.50-0.82)	0.82 (0.59-0.95)	0.59 (0.36-0.73)	0.65 (0.49-0.79)	0.73 (0.56-0.89)	0.59 (0.41-0.77)	0.66 (0.48-0.80)	0.70 (0.53-0.87)	0.64 (0.41-0.77)	0.55 (0.40-0.69)	0.51 (0.37-0.68)	0.60 (0.41-0.77)
Questioning patient credibility	0.45 (0.18-0.67)	0.46 (0.20-0.70)	0.46 (0.08-0.69)	0.56 (0.29-0.76)	0.60 (0.33-0.80)	0.54 (0.23-0.77)	0.46 (0.25-0.65)	0.47 (0.27-0.71)	0.47 (0.23-0.69)	0.60 (0.38-0.78)	0.71 (0.50-1.00)	0.54 (0.39-0.77)	0.52 (0.36-0.67)	0.42 (0.29-0.60)	0.69 (0.39-0.85)
Positive/preferred language	0.85 (0.79-0.90)	0.81 (0.74-0.88)	0.89 (0.80-0.94)	0.84 (0.79-0.89)	0.78 (0.71-0.85)	0.91 (0.82-0.96)	0.81 (0.75-0.87)	0.81 (0.74-0.88)	0.82 (0.73-0.89)	0.79 (0.72-0.85)	0.80 (0.73-0.88)	0.78 (0.68-0.85)	0.71 (0.63-0.78)	0.75 (0.67-0.83)	0.68 (0.57-0.77)
Macro average	0.73	0.77	0.71	0.75	0.77	0.75	0.68	0.72	0.66	0.67	0.73	0.62	0.56	0.57	0.58

	Enhanced model														
	ClinicalBERT			BERT base			SVM			Random Forest			Decision Trees		
	F1 (95% CI)	Precision (95% CI)	Recall (95% CI)	F1 (95% CI)	Precision (95% CI)	Recall (95% CI)	F1 (95% CI)	Precision (95% CI)	Recall (95% CI)	F1 (95% CI)	Precision (95% CI)	Recall (95% CI)	F1 (95% CI)	Precision (95% CI)	Recall (95% CI)
Marginalized language/identities	0.89 (0.85-0.93)	0.86 (0.80-0.91)	0.93 (0.87-0.96)	0.92 (0.88-0.96)	0.93 (0.88-0.97)	0.91 (0.84-0.95)	0.93 (0.89-0.97)	0.97 (0.94-1.00)	0.90 (0.84-0.95)	0.89 (0.84-0.94)	1.00 (1.00-1.00)	0.80 (0.73-0.88)	0.81 (0.74-0.86)	0.96 (0.91-1.00)	0.70 (0.61-0.77)
Difficult patient	0.78 (0.07-0.86)	0.79 (0.67-0.88)	0.77 (0.63-0.88)	0.73 (0.63-0.80)	0.65 (0.55-0.73)	0.83 (0.69-0.92)	0.63 (0.53-0.73)	0.64 (0.53-0.75)	0.63 (0.50-0.75)	0.59 (0.48-0.69)	0.60 (0.49-0.72)	0.59 (0.46-0.71)	0.51 (0.40-0.63)	0.54 (0.42-0.66)	0.50 (0.35-0.63)
Power/privilege	0.80 (0.65-0.90)	0.79 (0.62-0.91)	0.82 (0.59-0.91)	0.78 (0.61-0.89)	0.85 (0.68-1.00)	0.73 (0.45-0.86)	0.78 (0.64-0.91)	0.85 (0.70-0.97)	0.73 (0.59-0.91)	0.77 (0.61-0.88)	0.89 (0.75-1.00)	0.69 (0.50-0.86)	0.68 (0.50-0.81)	0.82 (0.65-1.00)	0.59 (0.36-0.73)
Unilateral/authoritarian	0.71 (0.53-0.84)	0.70 (0.53-0.86)	0.73 (0.48-0.86)	0.65 (0.46-0.80)	0.67 (0.47-0.86)	0.64 (0.36-0.82)	0.76 (0.62-0.88)	0.80 (0.67-0.95)	0.73 (0.50-0.86)	0.76 (0.62-0.88)	0.81 (0.65-0.94)	0.72 (0.50-0.86)	0.76 (0.62-0.87)	0.74 (0.60-0.90)	0.77 (0.64-0.96)
Questioning patient credibility	0.62 (0.39-0.80)	0.63 (0.38-0.83)	0.62 (0.31-0.77)	0.60 (0.40-0.77)	0.54 (0.33-0.73)	0.69 (0.31-0.85)	0.61 (0.42-0.80)	0.63 (0.44-0.88)	0.61 (0.31-0.77)	0.69 (0.51-0.86)	0.70 (0.52-0.91)	0.69 (0.46-0.85)	0.57 (0.38-0.74)	0.54 (0.37-0.77)	0.61 (0.39-0.77)
Positive/preferred language	0.89 (0.85-0.93)	0.86 (0.79-0.92)	0.94 (0.85-0.98)	0.91 (0.86-0.95)	0.93 (0.88-0.99)	0.89 (0.79-0.95)	0.85 (0.80-0.91)	0.82 (0.75-0.89)	0.89 (0.82-0.95)	0.84 (0.77-0.89)	0.84 (0.77-0.91)	0.83 (0.76-0.90)	0.77 (0.70-0.83)	0.76 (0.69-0.84)	0.78 (0.68-0.85)
Macro average	0.78	0.77	0.80	0.77	0.76	0.78	0.76	0.79	0.75	0.76	0.81	0.72	0.68	0.73	0.66

*Note.* Statistical significance test results between the initial and enhanced models can be seen in Table S1.

Abbreviations: BERT, Bidirectional Encoder Representations from Transformers; CI, confidence interval; SVM, Support Vector Machine.

We further examined the detailed performance scores of ClinicalBERT, which showed the best performances in the final enhanced models. The questioning patient credibility category showed the greatest improvement, with the F1-score increasing from 0.45 to 0.62, representing about 38% improvement. The marginalized language/identities and positive/preferred language categories, which achieved high F1-scores of 0.9 in the initial model, showed no improvement in the enhanced model. The other categories showed varied levels of improvement, with F1-scores increasing by 3% to 8% from the initial to the enhanced model.

## Discussion

In this study, we used various NLP models to identify stigmatizing and positive/preferred language in obstetric clinical notes. We employed traditional classifiers (SVM, Decision Trees, and Random Forest) and a transformer-based language models, ClinicalBERT and BERT base. We found that ClinicalBERT was the best performing model with an average F1-score of 0.78 in the final enhanced model. We also developed and employed semantic similarity-based search approach to increase the number of exemplars in training datasets in a less manually intensive way. Using this new approach, we were able to enhance the training dataset, which improved the performance of NLP models up to 21%. Previous research works on stigmatizing language have primarily used rule-based NLP approaches or simple NLP models that focus on keywords or term identification, which may limit identifying nuanced and comprehensive stigmatizing language categories accurately.^7^^,^^29^ Our study provides an important addition to advancing the field by showcasing the sophisticated capabilities of pre-trained and fine-tuned BERT models to capture nuanced and semantically meaningful language patterns.^20^

In the current study, ClinicalBERT and BERT base outperformed the 3 more traditional classification models. ClinicalBERT had the highest performance with an average F1-score of 0.78 in the final enhanced model. BERT models, with the self-attention mechanism, has a superior ability to handle complex language structures and effectively understands context and nuances in text.^20^ The self-attention mechanism is an important component of transformer-based models, such as BERT.^20^ This mechanism allows the model to weigh the importance of difference words in a sentence, which helps to better understand the context by focusing on relevant parts of the text.^20^^,^^30^ Another important factor contributing to ClinicalBERT’s superior performance is the initial pre-training of such models on a large corpus of text, which enables BERT models to quickly achieve good generalizability and performance on tasks, even with limited available training data.^20^ Thus, BERT models can be particularly advantageous at identifying stigmatizing language, which often involves subtle and context-dependent expressions that traditional classifiers might have missed. For example, difficult patient categories may have benefited from ClinicalBERT as it requires understanding context where a patient is subtly described as difficult. Furthermore, we found despite limited pre-training on clinical data, BERT base also performed well similar as ClinicalBERT. This may be because stigmatizing language is closely related to understanding social language and context, which aligns with previous study findings.^31^

Challenges of BERT models include the higher computational cost associated with transformer-based models, which require significant processing power and memory.^32^^,^^33^ Additionally, the complexity of BERT models can contribute to longer training times and the need for more extensive hyperparameter tuning compared to traditional classifiers.^34^ Despite these challenges, the benefits of using BERT in accurately identifying subtle and nuanced stigmatizing language patterns in clinical notes were evident in the current study. Our NLP methods offer a promising opportunity for future research to advance the identification of stigmatizing language in obstetric settings. Future studies could build on this work by conducting external validations with new datasets from diverse geographic locations and varied clinical settings to establish robustness and generalizability.

We also examined how performances of NLP models improve by employing a streamlined approach based on semantic similarity to search and extract additional exemplars in clinical notes that can be used for model training. This approach effectively enhanced the performance by 7% to 21% in F1-scores across different NLP models. This approach is less labor-intensive than traditional human annotation because it can automatically search and extract semantically similar exemplars with the already annotated gold standard dataset. Thus, it is an efficient and practical solution to address challenges around having scarce exemplars following initial human annotation^15^ by expanding and enriching the initial training dataset with new, contextually relevant exemplars. Importantly, this approach still required manual human verification of the extracted exemplars. This is an important step to ensure the extracted exemplars accurately represented the intended language categories given that cosine similarity metric can result in arbitrary semantic similarities where identified candidates reflect low or meaningless similarities with the queries.^35^ While our approach was substantially less labor-intensive than fully annotating new data manually and effective in enhancing the performances of NLP models, eliminating the need for human efforts in the annotation process remains a challenging part of NLP work.

Even after such enhancement, further improvement is needed to accurately identify certain categories of stigmatizing language. For instance, the questioning patient credibility category consistently showed lower F1-scores, ranging from 0.57 to 0.69 in the enhanced models across different NLP models. Potential reasons for this include the low number of positive case exemplars (fewer than 100 cases identified in the enhanced model) and the highly nuanced nature of this category. During the initial human annotation process, expert annotators noted that questioning patient credibility is not as explicit or straightforward as other categories, often requiring a deep understanding of contextual meanings and subtle expressions unique to each situation. The following exemplars showcase the complexities of accurately interpret the subtle meaning of the text: “Pt [patient] does not think she has gestational diabetes. She states she ate very sweet rice (a dish from her country) the day before the glucose challenge which is what she believes this is the reason for the high value. states it has never been high before,” and “reports daily palpitations and atypical chest pain/SOB [shortness of breath] but able to walk several city blocks without issue.” This contrasts with more straightforward categories with common identifiable keywords frequently appearing in the clinical notes. For example, categories like marginalized language/identities often include explicit terms such as “obese” or “abuse,” which are easier to recognize consistently. These high-signaling keywords may be the reason why SVM performed better than ClinicalBERT for marginalized language/identities category. Further refinement of deep learning models that can better capture nuanced and context-specific language categories is needed in future research.

Importantly, identifying stigmatizing language in clinical notes is an emerging field of research that is highly nuanced and somewhat subjective. For example, language that can be interpreted as stigmatizing for some readers (eg, “she does not use drugs or smoke but drinks 6x/week” or “financially supports self”) may reflect objective assessments to other readers. It may also reflect standard and common language that are often used in clinical documentation without negative connotations (eg, “denies” or “complaints”). Although we cannot discern the true intention or sentiments of writers in the clinical documentation, we conducted rigorous qualitative analysis^12^ informed by previous research in this field^14^ to improve the rigor of our language categories. We also had a multidisciplinary team of experts, including obstetric and gynecology physicians, nurses, and nonclinical researchers, such as data scientists, to bring broader perspectives and further enhance the rigor and trustworthiness of our qualitative work.

For instance, we found that information such as “financially supports self” is not routinely documented in clinical notes for all patients. Including such information can inadvertently highlight or imply a socioeconomic status that may not be relevant to clinical care, potentially stigmatizing certain groups. This is particularly significant for individuals who might be perceived as belonging to a lower socioeconomic group, explicitly or implicitly through factors like appearance, language proficiency, or insurance status. Thus, the nuance in identifying stigmatizing language is important, as the patient may read their own notes and interpret this language in a negative way, or future clinicians who read the previous note may transmit implicit or explicit bias into their care of the patient. Although the language we identified may not appear stigmatizing on its own, when this type of language is applied more frequently to people with marginalized identities, it may reinforce implicit biases carried by clinicians. It is recommended to practice clinical documentation that is centered around the patient’s experience and circumstances to eliminate potential stigma and bias. For example, stating “patient reports drinking 6x/week” may introduce assumption or bias that the patient drinks 6-7 drinks a day, 6 days a week. In this case, providing additional contextual details such as “patient reports drinking 6x/week, one beer per day” or reasons why their drinking increased due to personal life events would be important to reduce such assumption or bias.

Additionally, positive language, such as “patient desires epidural,” could also reflect the writer perceiving patient as demanding to some readers. We identified “desires” as positive/preferred language based on previous literature^14^ and our rigorous qualitative analysis.^12^ We determined that, though we cannot know the true intention of the write, such positive language contrasts from other language that are more stigmatizing (eg, complaints). Future research can explore healthcare user perspectives around stigmatizing language to further improve the subjective nature of stigmatizing language. Healthcare users in birth settings can read their own notes and identify what they perceive as stigmatizing regardless of writers’ intention to use such language in clinical notes.

The findings of this study have significant clinical implications. One of the primary implications is the potential application of NLP models for real-time monitoring of clinical notes to automatically detect instances of stigmatizing language and re-iteration of biasing, unnecessary patient descriptors as they are being recorded. This can enable automatic alerts or prompts to clinicians and healthcare team members to modify their language to be more respectful. Additionally, applications could recommend an alternative, non-stigmatizing language clinicians can use. This approach can reduce the potential transmission of bias throughout the clinical team. For example, using language that implies a patient is “difficult” can give the next clinician a biased impression of the patient by reading the chart before even seeing them, which can impact the care delivered. Modern-day patients have quick and easy access to read their clinical notes and about at least 1 in 10 patients felt judged and/or offended by the language they see in clinical notes.^36^ Real-time monitoring and alerts would prevent such stigmatizing language from being recorded, contributing to supportive and non-stigmatizing care and improved patient experiences for all.

To further advance our work and the field of research in stigmatizing language, future studies should explore the differences in the use of stigmatizing language in different racial and ethnic subgroups. For instance, previous studies have found that notes containing stigmatizing language were more likely to be found in non-Hispanic Black individuals compared to non-Hispanic White individuals among patients with health conditions such as diabetes and chronic pain.^37^^,^^38^ Understanding similarities and differences in types and frequencies of stigmatizing language found in obstetric clinical notes can potentially reveal underlying biases and disparities in healthcare, particularly around communication and documentation patterns for minoritized individuals, which have not been investigated in previous research. Including diverse community members and experts is important to further refine the language categories in a way that reflects nuanced differences in future research. We plan to incorporate this approach in our ongoing and future studies to ensure the language categories are comprehensive and culturally sensitive.

Another important area for future research is to examine how stigmatizing language is associated with the quality of obstetric care and health outcomes. Studies found greater diabetes severity is associated with a higher probability of having stigmatizing language documented in clinical notes.^37^ In contrast, usage of non-stigmatizing language is associated with better follow-up care and referrals among patients who have opioid use disorder.^39^ Therefore, there is a strong potential that documentation of stigmatizing language may be associated with obstetric care quality and health outcomes, which should be further invested in future research. Our planned future studies aim to investigate these areas to provide further insights in reducing bias and disparities in healthcare for the perinatal population.

### Limitations

The current study has several limitations. First, this study was conducted in one geographic location, a large metropolitan city in the Northeast United States. Therefore, findings may not generalize to areas with substantially different geographic and demographic characteristics. We obtained EHR data from the birth admission only, and the use of stigmatizing and language in outpatient obstetric settings remains unexplored. Future research in diverse geographic and healthcare settings may be needed to validate the generalizability of findings and provide further insights into stigmatizing language in a more comprehensive care setting. Second, our NLP models were built based on our specific language categories and may not be apply to new or different stigmatizing or non-stigmatizing language categories. Third, we used TF-IDF for feature extraction and selection, which is widely used for NLP, but has some inherent limitations. TF-IDF examines how often words appear in a document compared to how often they appear across all documents. Although this helps highlighting important words, it is limited in understanding the context in which words are used. As a result, TF-IDF may have difficulty capturing more nuanced semantic meanings and understanding contextual relationships within the text. Future studies may consider using more advanced feature extraction techniques such as contextual embeddings to better capture semantic meanings. Fourth, despite our various preprocessing steps, separating clinical notes into sentences remains challenging, potentially limiting the NLP model performance. Lastly, we observed lower interrater reliability in creating the initial human-annotated dataset. The agreement rate among the annotators was 72% and Cohen’s Kappa was 0.4, indicating fair agreement.^40^ Several factors could contribute to this, including the subjectivity involved in interpreting clinical notes and language uses as well as complexity and nuanced nature of stigmatizing language. To ensure the quality and accuracy, we spent extensive effort and time to discuss any discrepancies among annotators to reach a consensus. The focus of annotation in computational linguistics is its usefulness for further computational processes, meaning that annotated data are still valuable even if interrater reliability is not high.^41^ In addition, deep learning models, such as BERT, can robustly handle noisy data with less reliable annotations and perform well.^42^ This robustness ensures that our final ClinicalBERT model can effectively learn from the provided data despite potential limitations.

## Conclusion

The current study provides advancement in identifying stigmatizing language in obstetric clinical notes by employing an advanced transformer-based language model, ClinicalBERT. Our approach based on semantic similarity to automatically search to extract additional exemplars for model training enhanced the performance while reducing the burden of human annotation. ClinicalBERT effectively captured nuanced and context-dependent stigmatizing language patterns in obstetric clinical notes, highlighting potential for real-time monitoring and alerts to prevent stigmatizing language use and to reduce bias in obstetric settings. Future research should explore stigmatizing language in diverse environments, including varied geographic locations and clinical settings, to contribute to high-quality and equitable care for the perinatal population.

## Supplementary Material

ocae290_Supplementary_Data

## Data Availability

Data are not available for public access due to the limitations imposed by the IRB protocol.
