# Supplementary material for: Identifying stigmatizing and positive/preferred language in obstetric clinical notes using natural language processing
Source: J Am Med Inform Assoc. 2024 Nov 21;32(2):308–17. doi: 10.1093/jamia/ocae290 (PMC11756426; doi:10.1093/jamia/ocae290)
Supplement: ocae290_Supplementary_Data [file ocae290_supplementary_data.docx]

**Supplementary**

Table 1. Statistical significance test between initial and enhanced models

| **Performance Metric** | **Initial Model** | **Enhanced Model** | **W-statistic** | **p-value** |
| --- | --- | --- | --- | --- |
| F1 | Clinical BERT | Clinical BERT | 1 | **0.0313** |
| Precision | Clinical BERT | Clinical BERT | 13 | 0.9375 |
| Recall | Clinical BERT | Clinical BERT | 3 | 0.0781 |
| F1 | BERT base | BERT base | 8 | 0.375 |
| Precision | BERT base | BERT base | 13 | 0.9375 |
| Recall | BERT base | BERT base | 10 | 0.5781 |
| F1 | SVM | SVM | 1 | **0.0313** |
| Precision | SVM | SVM | 5 | 0.1563 |
| Recall | SVM | SVM | 0 | **0.0156** |
| F1 | Random Forest | Random Forest | 0 | **0.0156** |
| Precision | Random Forest | Random Forest | 1 | **0.0313** |
| Recall | Random Forest | Random Forest | 0 | **0.0156** |
| F1 | Decision Trees | Decision Trees | 0 | **0.0156** |
| Precision | Decision Trees | Decision Trees | 0 | **0.0156** |
| Recall | Decision Trees | Decision Trees | 4 | 0.1094 |

*Note.* Performance metrics for each language category from the initial and enhanced models were used as a paired unit of measure. The differences between the performance metrics from the initial and enhanced models were analyzed using the Wilcoxon Signed Ranked Test.
